# Process based quality improvement using a continuous renal replacement therapy dashboard

**DOI:** 10.1186/s12882-018-1195-8

**Published:** 2019-01-11

**Authors:** Theresa A. Mottes, Stuart L. Goldstein, Rajit K. Basu

**Affiliations:** 1 Renal Section, Department of Pediatrics, Texas Children’s Hospital, Baylor College of Medicine, Feigin Building ,1102 Bates Ave, Suite 245, Houston, TX 77030 USA; 2 Center for Acute Care Nephrology, Cincinnati Children’s Hospital Medical Center, University of Cincinnati College of Medicine, Cincinnati, OH 45229 USA; 3 Division of Critical Care Medicine, Children’s Healthcare of Atlanta, Emory University, Atlanta, GA 30322 USA

## Abstract

**Background:**

The prevalence of continuous renal replacement therapy (CRRT) utilization in critically ill patients with acute kidney is increasing. In comparison to published and on-going trials attempting to answer questions surrounding the optimal timing of CRRT initiation, anticoagulation, and modality, a paucity of literature describes the quality of the therapy delivered.

**Methods:**

We conducted a single-center process improvement project to determine if a methodology to assess the quality of CRRT delivery could lead to improvement in CRRT delivery outcomes. We developed three broad categories of objective CRRT metrics to assess longitudinally, enabling creation of a CRRT Dashboard. Following the objective categories of “filter”, “prescription”, and “fluid balance” over time allowed us to perform quarterly analyses, target provider based CRRT education, and address variation from our standard of care. From 2012 to 2017, 184 critically ill patients received CRRT.

**Results:**

We report a mean filter life of 56 + 28.4 h, a 60-h filter life of 62%, and unplanned filter changes of 15%. Compared to a minimum target prescription of 2000 ml/1.73 m2/hour, we report the mean prescribed dose (2300 ml/1.73 m2/hour) and the rate of patients receiving at least the minimum prescription (98%). Finally, using a 10% deviation in the acceptable range of desired daily patient fluid balance, we report 83% CRRT patient days achieving an acceptable stipulated fluid goal.

**Conclusion:**

We report the implementation of a quality dashboard and adopting quality improvement strategies provided a platform for measuring adherence to our institutional standards and the delivery of CRRT, specifically on the process of the care.

## Background

Acute kidney injury (AKI) occurs commonly in critically ill patients [1]. The current recommended management of AKI consists of supportive care including optimization of hemodynamics and systemic oxygenation with limitation or removal of ongoing iatrogenic sources of renal injury (e.g., nephrotoxic medications). For patients with severe AKI associated with significant electrolyte abnormalities and/or oliguria and concomitant fluid accumulation, continuous renal replacement therapy (CRRT) is often utilized for stabilization [2]. The increasing prevalence of CRRT utilization parallels recent epidemiologic data demonstrating the increase in AKI prevalence across the world in both adults and children [3].

CRRT is an invasive and technically complex form of extracorporeal support. Similar to other forms of invasive organ support such as mechanical ventilation and extracorporeal membrane oxygenation (ECMO), the majority of existing data and ongoing research investigate the application of the therapy with patient outcomes such as length of stay, complication rates, and mortality [4]. Also, just as in mechanical ventilation and ECMO, controversy surrounds the application of CRRT and a majority of research investigates aspects such as patient selection, timing of initiation, CRRT modality, anticoagulation method, and dose [5, 6]. The processes by which CRRT is delivered and the quality of that delivery, however, lack formal consensus for a standard of care. Compared to the controversies listed above, very little published data exist describing how the therapy is actually delivered and/or how well the actual delivery matches the goals set by the prescribing provider(s) [7]. The assessment of CRRT quality represents a clinical gap of knowledge.

Optimal CRRT delivery requires a multidisciplinary, simultaneous and continuous coordination of care from multiple providers and areas of expertise. The complexity can lead to significant deviation in how CRRT is delivered between providers and institutions. The deviation can subsequently lead to significant discrepancies in CRRT delivery which ultimately can affect the actual receipt of the therapy by the patient. Few protocols are published describing how services can be coordinated to streamline CRRT delivery. Currently, there is a lack of data and benchmarks to evaluate the process of CRRT delivery that would enable comparisons between patient populations, providers, or institutions. Studying the care delivery process is crucial and can be performed by measuring and constantly surveilling process and outcome metrics. The study of process measures can render an index of quality by providing a quantifiable level of adherence to accepted performance standards. Incorporation of quality improvement science in critical illness is becoming more common and an increasing level of scrutiny is now given to level of variability from standards of care [8].

**Fig. 1:**
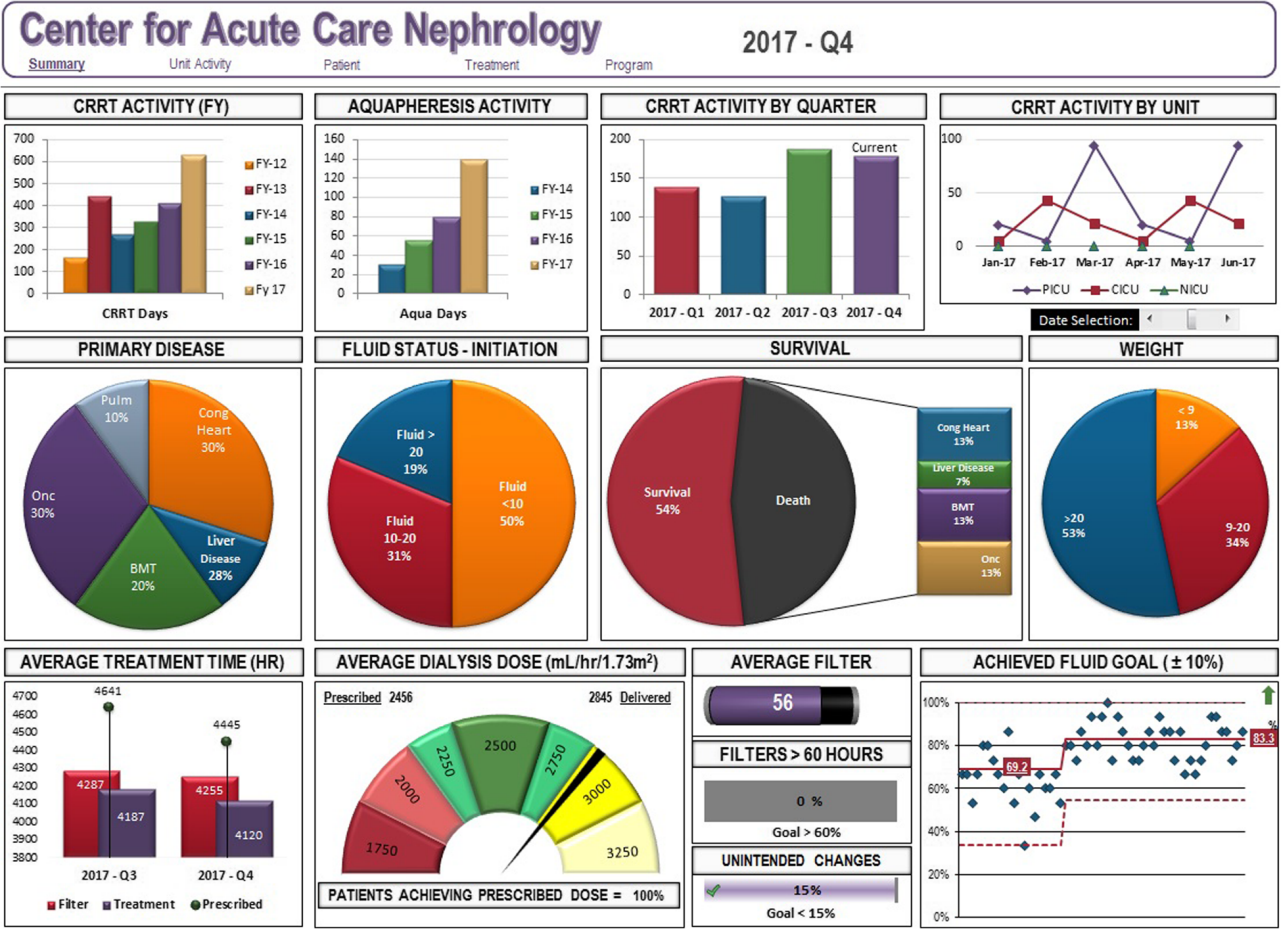
Dashboard for Delivery of Renal Replacement Therapy. A sample dashboard is shown in this figure, created as a composite of the metrics followed to assess the quality of renal replacement therapy (RRT) delivery at our institution. Continuous RRT (CRRT) activity is shown on the top line of graphs, broken down by fiscal year (FY), aquapheresis, quarter of the year, and intensive care unit (ICU) environment (P = pediatric, C = cardiac, N = neonatal). The second line of displayed data are all pie-chart and visually give a cumulative indicator of primary patient disease, percent fluid accumulation at time of RRT initiation, survival percentage, and starting weight of the patients receiving RRT. The final line of the dashboard displays data on performance and delivery: treatment time, dialysis dose and relationship of prescribed versus delivered, filter lifespan and % of filters above our institutional “baseline”, unintended filter changes, and finally the achievement of fluid goals as a function of time (in FY and quarter)

We hypothesized commonly used variables related to CRRT delivery could yield process and outcome metrics that can be tracked longitudinally. We aim to create a set of standards suitable for benchmarking, comparisons within our program as well as other programs, and for the purpose of creating a standard of care for adherence when delivering CRRT. This report describes the development of a CRRT Dashboard as a quality improvement tool (Fig. 1).

## Methods

### Patients

All data were collected from intensive care units (newborn, cardiac, and pediatric – denoted NICU, CICU, and PICU respectively) from Cincinnati Children’s Hospital Medical Center. The data are collected from a longitudinal assessment of CRRT delivery. Time windows for data collection are described in each section of the results per metric of interest, but the overall time of study for the dashboard reported here is from July 2012 to June 2017. This project was approved by the CCHMC Institutional Review Board with a waiver of informed patient consent as this was a non-interventional quality improvement project. In the data shown, the timeframe years is denoted as fiscal year (FY) (as opposed to calendar year). For example, FY 16 is the time frame of July 2015 through June 2016. The number of patients studied is denoted per metric quantified. CRRT patient days are calculated as the sum total of days CRRT used for the total number of patients over a given time window.

### Dashboard metrics

#### Activity

Activity metrics are updated on a quarterly and yearly basis. The following metrics are tracked: 1) *RRT Activity* – a total number of RRT days accounting for continuous RRT (CRRT), 2) CRRT Activity by Unit – total number of CRRT patient days separated by unit, 3) Primary Disease of patients receiving CRRT (classified by major organ system of pathology), 4) Patient ICU admission weight – patients categorized into categories: < 9 kg, 9-20 kg, and > 20 kg, and 5) Survival broken down by primary organ system pathology.

#### Filter survival

The optimal delivery of CRRT is contingent on maintaining a well-functioning CRRT circuit, thereby minimizing the time the patient is not receiving therapy. The time a patient is receiving therapy can be derived from the functional survival of the filter on a given machine. Filter survival, must be adjudicated however, as it is influenced by prescription, providers, and the characteristics of the patient. Filter survival is therefore assessed in two ways: “filter life” and “unplanned filter changes” (UFCs).

*Filter life* is defined as the duration of time, measured in hours, an individual filter or circuit is delivering therapy to the patient. Filter life is the difference between start time of the therapy and end time. Filter life can be objectively measured in two ways. Individual filter life is the duration of time a single filter functions for a single patient. Groups of filters are measured as percentage of filters surviving to a period of time (in hours). An a priori cut-off of 60 h was used as the operating benchmark of comparison for filter life [9].

*Unplanned Filter Changes* provide an objective measure of variation that evaluates the whole CRRT delivery system. Filter changes were divided into planned and unplanned (PFC and UFC, respectively). Any filter changed after 60 h or changed before 60 h because of a patient procedure (e.g. travel to the operating room, an emergent patient event, or patient death was denoted as a PFC [10]). Any filter change prior to 60 h exclusive of the reasons listed below is denoted as UFC. Filter changes were studied in groups of 15 consecutive filters. UFC percentage was calculated by counting the number of UFCs by the total number of filter changes (PFCs + UFCs).

#### Prescription

Prescribed and achieved CRRT effluent doses were analyzed. Both values were measured for individual patients and groups of patients.

*Discrepancy* between prescribed and delivered was quantified by simultaneously measuring both values and calculating % delivered (i.e., the number of patients achieving delivered dose divided by total number of patients in the specific period).

*Minimum Prescription*: Using the total number of CRRT hours delivered to the patient and the total effluent measured in mL, the delivered CRRT dose in mL/kg per hour as well as mL/1.73 m^2^ (patient body surface area) per hour were calculated. The median delivered dose for patients over a given quarter was analyzed. A minimum baseline prescription of 2000 ml/1.73 m^2^/ hour was used as the “red” zone. An odometer style gauge was used to visualize this metric.

*Average Treatment Time* is the quantified average of time for an individual patient treatment course on CRRT. The dashboard displays average life of filter, average prescribed time, and average filter time (in hours).

#### Fluid balance

Fluid accumulation is objectively measured by a previously developed derived formula [11] in the context of RRT utilization. Fluid accumulation is expressed as percent fluid overload (% FO). Fluid as a metric on the dashboard is separated into fluid status at initiation and achievement of daily fluid goals. The formula for calculating fluid overload is:

[((Intake (liters) from ICU admission to CRRT start - Output (liters) from ICU admission to CRRT start)/1000))/ICU admission weight (kg)] [12].

*Fluid Status at Initiation* is stratified into categories: less than 10, 10–20%, and greater than 20%. The categories were developed utilizing the literature and associated outcomes [11]. We track both the % FO at the time of nephrology consultation and the % FO at the time of RRT initiation.

*Achieved Fluid Goal (Desired Total Fluid Output)*: The Nephrology and Critical Care team at our institution agree upon a daily fluid balance goal for all patients receiving CRRT during morning rounds. The a priori designated acceptable range of a fluid goal is ±10% of target. Calculation of the variability from the target fluid goal assumes the actual 24-h total output will be equivalent to the total 24-h intake minus the net 24-h fluid balance goal. The percentage of patients shown in the dashboard control chart is the percentage achieving the acceptable range of ±10% of the desired fluid output. For example, a patient with a 24 h total intake of 2200 mL and 24 h net fluid goal of a negative 250 mL, has desired total output of 2450 mL. If the actual patient total output for the same 24 h period is 2350 mL, the patient has reached 95% of the desired total fluid output, achieving the acceptable range of ±10% of the desired fluid balance. “Desired fluid output” was chosen as the metric to follow as “net fluid balance” results in the possibility of a failure to calculate achievement based on a denominator of “zero”. *Statistics:* Individual patient or filter data are displayed on the dashboard. Variables with normal distribution were compared using the Student’s T-test and Fisher’s exact tests of comparison with ANOVA for multiple comparisons. Non-parametric data were compared using the Mann-Whitney U test. Circuit survival data were censored for Kaplan-Meier analysis for the following indications: patient death or withdrawal of support, patient procedure requiring CRRT discontinuation, patient regaining renal function or transition to intermittent hemodialysis and scheduled circuit change [9, 13]. A *p*-value for < 0.05 was considered significant for these comparisons. Data analysis was performed using SAS software 9.4, Cary, NC, USA, (Copyright © 2002–2012 SAS Institute Inc).

Longitudinal evaluation of dashboard metrics was conducted using statistical process control method to identify changes from baseline rates for each metric [14]. We evaluated all rates per 15 CRRT filters used. We set an a priori standard of eight consecutive metric rates above or below the baseline rate to qualify as a statistical change, (or special cause in process control vernacular), which corresponds to 99.7% likelihood that the change observed resulted from the improvement intervention [15]. This methodology has served as the primary quality improvement assessment measurement to track the serious safety event rates for the past 15 years at CCHMC [16] .The control chart facilitates the monitoring of periodic changes using special-cause variation rules. Visualization of the trends subsequently can lead to root-cause analyses of variability, highlight weaknesses or errors, and the effect of interventions.

## Results

For the purpose of this analysis, data analyzed were from FY2012 through FY2017 (analyzed either per quarter, per annum, or longer depending on the metric of interest). Quarterly “CRRT Dashboards” are created by extracting data from three month blocks in that period of time (Fig. 1).

### RRT activity

One hundred eighty-four patients received RRT between 2012 and 2017. CRRT was delivered for a total of 2090 days (1221 PICU, 535 CICU, 63 NICU). CRRT was utilized in patients with a wide variety of primary pathologies and roughly half (53%) weighed more than 20 kg. Eighty-seven (47%) patients did not survive to ICU discharge.

### Filter life and unplanned filter changes

Data assessing filter life are compiled from 2013 to 2017 and shown in Table 1. All patients received CRRT using the Prismaflex™ (Gambro Renal Products, Lund, Sweden). Per manufacturer recommendations, filters are changed every 72 h. The mean filter life was 56 ± 28.4 h. Sixty-two percent of filters were functional at 60 h. Unplanned Filter Changes steadily decreased from 40% in 2014 to below 15% in 2017.

**Table 1:** Filter Life

Time Period	2013 Q1/Q2	2013 Q3/Q4	2014 Q1/Q2	2014 Q3/Q4	2015 Q1/Q2	2015 Q3/Q4	2016 Q1/Q2	2016 Q3/Q4	2017 Q1	2017 Q2	2017 Q3	2017 Q4
Mean Filter Life (hours)	ND	50	47	44	44	53	54	55	43	45	53	56
Unplanned Filter Change (%)	33	40	40	34	34	34	26	16	40	26	15	15
Filter Survival > 60 h (%)	58	58	64	62	58	58	67	58	49	56	79	78

### Prescribed effluent dose

The mean prescribed CRRT dose was 2421 ml + 259 /1.73m^2^/hour. Our data demonstrate a prescribed dose consistent with our institutional standard during this period (Table 2). Furthermore, 100% of prescriptions were above the minimal target dose of 2000 ml/1.73 m2/hour.

**Table 2:** Dose of Continuous Renal Replacement Therapy

Time Period	2013 Q1/Q2	2013 Q3/Q4	2014 Q1/Q2	2014 Q3/Q4	2015 Q1/Q2	2015 Q3/Q4	2016 Q1/Q2	2016 Q3/Q4	2017 Q1	2017 Q2	2017 Q3	2017 Q4
Mean Prescribed (mL/hr./1.73 m^2^)	2200	2400	3080	2323	2308	2368	2390	2360	2768	2133	2270	2456
Mean Delivered (mL/hr./1.73 m^2^)	ND	2400	3900	3040	3066	2879	2838	3132	3167	2484	2770	2845

### Delivered vs. prescribed effluent dose

The rate of patients achieving at least 90% of the prescribed dose increased from 2014 to 2017 (87 to 100%) and remained at 100% for all subsequent quarters.

### Fluid balance

One-half of the 184 patients studied in our dashboard initiated CRRT at a fluid overload < 10%. The remaining 50% were greater than 10% fluid overload, further categorized to 10–20% (57 patients) and > 20% (35 patients). We observed an increase in the achievement of daily desired fluid balance + 10% goal from 69.2 to 83.3% which has persisted for the last 8 consecutive quarters.

## Discussion

Since the landmark report by the Institute of Medicine *To Err is Human: Building a Safer Health System*, a significant amount of human and financial capital has been dedicated to improve patient safety [17]. A central conclusion from the report indicated the root of medical errors were in part secondary to a high degree of practice variability and inconsistency. Since that time, practice variation within and between institutions has become a focus of study in quality improvement [18, 19]. Reducing practice variation, and creation of care standards has ultimately lead to an increase the level of quality and safety for patients. Significant practice variation has already been shown in multiple patient environments to be directly associated with poor patient outcome [19]. In this report, we describe the creation of an acute RRT dashboard with numerous quantifiable outcome metrics to assess and in some cases, track improvement in the quality of care delivered.

The dashboard provides an ongoing assessment of performance and facilitates analyses of variations and deviations from standards of care. For example, filter survival is a litmus test for a well-functioning circuit and can therefore be used as a reflection of CRRT quality and real-time maintenance [20]. By a time measure, filter life varies greatly in the existing literature from 1 to 188 h [9, 20]. A pediatric multicenter prospective observational study studying 138 patients reported a mean filter life of 44.7 ± 35.9 h [9]. By survival percentage, 70% of filters were functional at 60 h [9]. We therefore incorporated 60 h as our accepted process standard for filter life. Although the median filter life is important to follow over time, unplanned filter changes reflect subtler RRT practice patterns and provide an objective measure of variation in the CRRT delivery system. Existing data indicate insufficient dose (< 20 ml/kg/hr. in adults) to be unsafe and result in poor outcomes [21]. Additionally, existing data indicate that actual dose delivered can be significantly different from the dose prescribed [22]. In practice, the most significant factor affecting delivered dose is time off-filter; single-center study reports a median downtime of 3.0 h (1.0–8.3 h) per day [23]. The most common causes of time off filter or “down-time” are filter clotting, catheter malfunction, machine alarm and procedures [24].

Visualization of the trends subsequently can lead to root-cause analyses of variability, highlight weaknesses or errors (e.g., inadequate anticoagulation or prescription, staff inexperience), and the effect of interventions (e.g., introduction of protocols, education sessions for staff) [10, 25, 26].

**Fig. 2:**
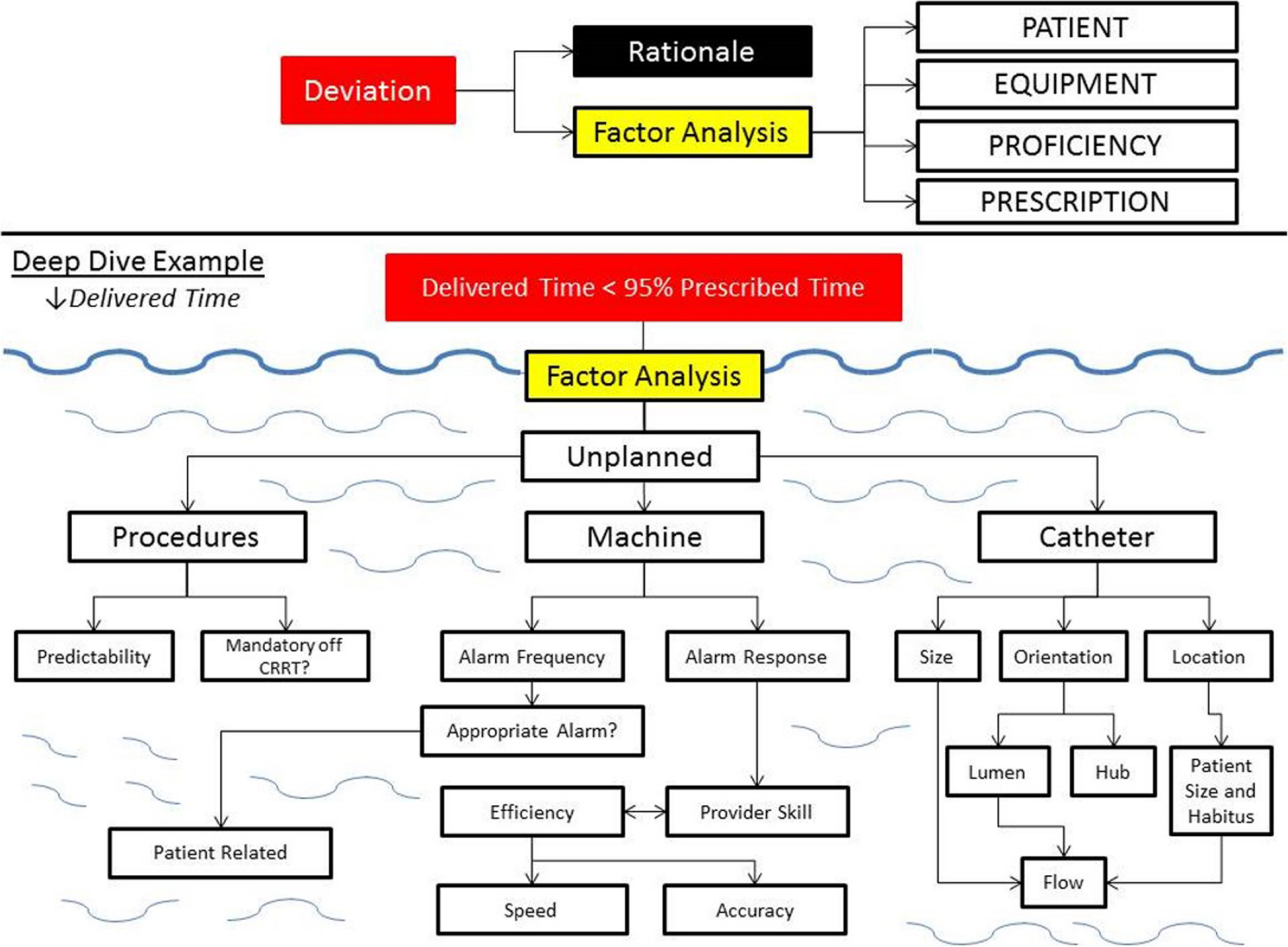
Deep Dive for Practice Deviation. On tracking data, the detection of a deviation from our institutional normative values triggers a “deep dive” factor analysis. The rationale (if present) for the deviation is determined and then individual factors are studied relating to patient, equipment, and the convergence of the two with the provider team (i.e., technical proficiency and prescription). The example shown is a standard deep dive list of questions asked for a deviation in delivered versus prescribed time

Upon detection of a deviation, our institutional practice is to investigate for the potential root cause. We term this investigation a “deep dive” (Fig. 2), which constitutes a factor analysis of patient (selection criteria, initiation, size and body habitus, special circumstances), equipment (inclusive of access catheter and machine), technical proficiency (nursing care and pharmacy), and the prescription of the therapy (modality, dose, anticoagulation). The example shown in Fig. 2 is a standard starting point to investigate a deviation in delivered time vs. prescribed time. A deviation was noted in Q1 FY 2017 (Tables 1-3). At this time, the deep dive discovered a single patient averaged 20.2 h of therapy per day, with a filter life approximately 18 h. This resulted in significant downtime, dramatically influencing the delivered CRRT dose. Further examination revealed the circuit life was being compromised by the excessive circuit clotting which can be predicted by a high pressure drop across the filter. To ensure adherence to our institutional anticoagulation standards, we analyzed our post-filter ionized calcium levels. We confirmed that the majority of the levels were within adequate range and the adjustments to the anticoagulation rates were according to the protocol. Through the deep dive we were able to confirm adherence to the standard practice guideline with regards to the prescription and anticoagulation protocols and no process corrective action was necessary. In Q2 FY2017, the CRRT trained nurses from the cardiac intensive care unit all came through our RRT educational program for the second time. The resulting effects of these actions were apparent in the data that emerged in Q3 and Q4 FY2017.

**Table 3:** Continuous Renal Replacement Therapy Treatment Times

Time Period	2014 Q1/Q2	2014 Q3/Q4	2015 Q1/Q2	2015 Q3/Q4	2016 Q1/Q2	2016 Q3/Q4	2017 Q1	2017 Q2	2017 Q3	2017 Q4
Mean Prescribed Time (hours)	185	210	278	4511	4060	4916	2559	2915	4641	4445
Mean Filter Time (hours)	160	176	199	3815	3825	4408	2176	2683	4287	4255
Mean Treatment Time (hours)	150	170	190	3695	3529	4262	2024	2618	4187	4120
Delivered Time (%)	81.1%	81.0%	68.3%	81.9%	86.9%	86.7%	79.1%	89.8%	90.2%	92.7%

In pediatric practice, perhaps the most common indication for acute RRT initiation is excessive fluid overload and oliguric AKI [27]. The Prospective Pediatric CRRT (ppCRRT) registry introduced the concept of quantifiable fluid overload and in retrospective study described how incremental escalation in fluid overload at the time of RRT initiation carried a direct, increasing association with patient mortality, independent of severity of illness. A recent meta-analysis of available pediatric data concludes that significant fluid overload is associated with poor patient outcomes [28]. Of note, regulation of patient fluid balance is a *modifiable* aspect of critical illness, regardless of the presence of kidney injury. Regular and consistent adjudication of both fluid administration and fluid removal, accounting for the phase of illness and context of the patient, can be crucial to patient outcome [29]. Of note, pediatric and neonatal patients may be particularly affected by fluid dysregulation given the marked heterogeneity in total body water percentage as a function of age. In practice, the desire to mitigate fluid accumulation and to remove fluid is a common indication for CRRT, therefore measuring the percentage of fluid overload at the start of CRRT is an essential measure. Precise control over fluid balance is a primary benefit and common indication of CRRT utilization in the management of critically ill patients; tracking the efficacy of attaining a fluid “goal” as a discrete metric is both obvious and possible. While variations exist in how fluid removal rates are ordered and implemented (e.g., hourly, per shift, or per 24-h period) attention to the achievement of fluid balance targets and factors influencing those targets is vital.

**Fig. 3:**
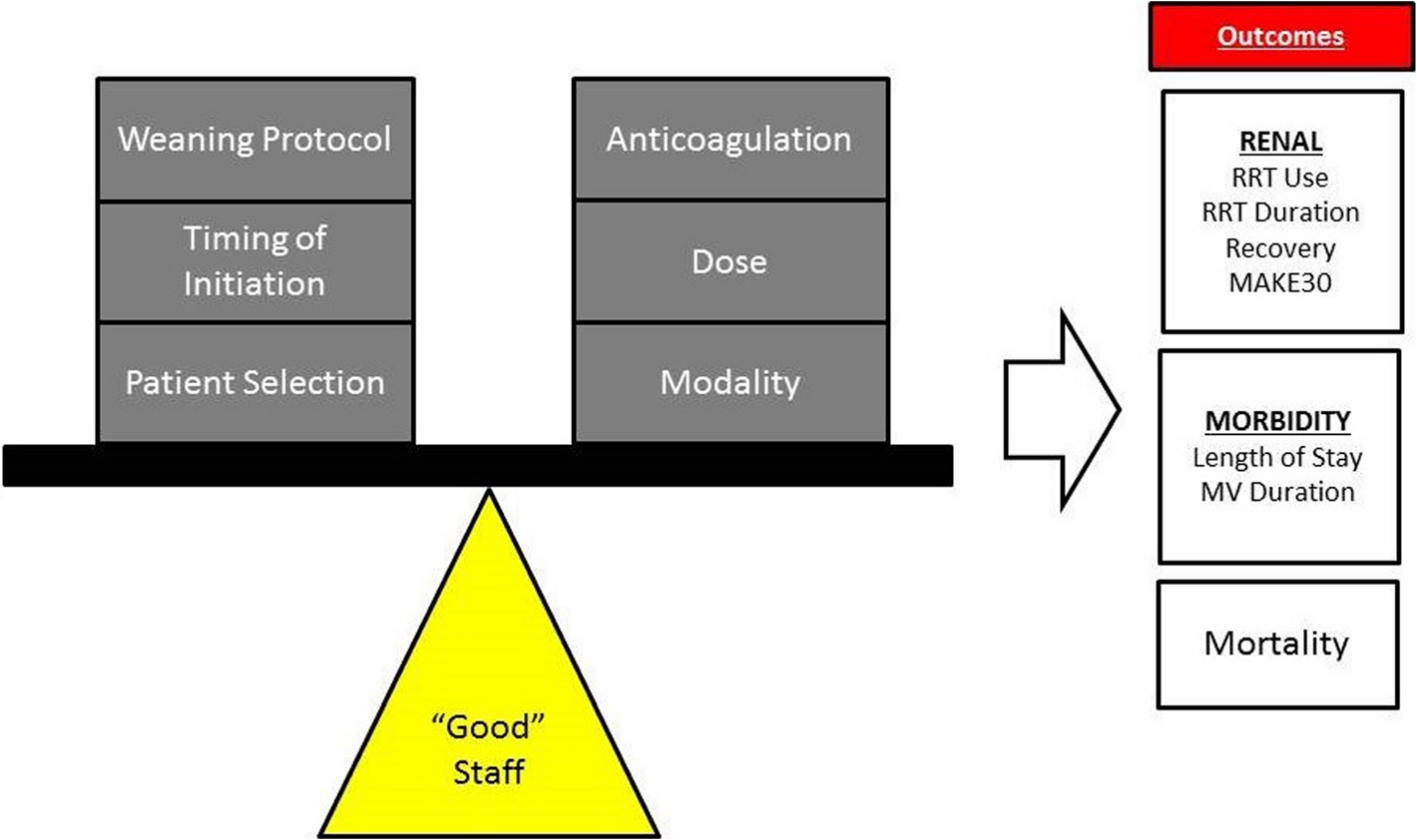
Controversies in Continuous RRT. In the existing paradigm, the majority of prospective studies investigating RRT attempt to answer the questions of which aspect of RRT is most important and how they balance each other to affect patient outcomes. Each of the aspects would assume an arbitrary level of “acceptable” staff performance. (RRT = renal replacement therapy, MAKE30 = major adverse kidney events in 30 days, MV = mechanical ventilation)

**Fig. 4:**
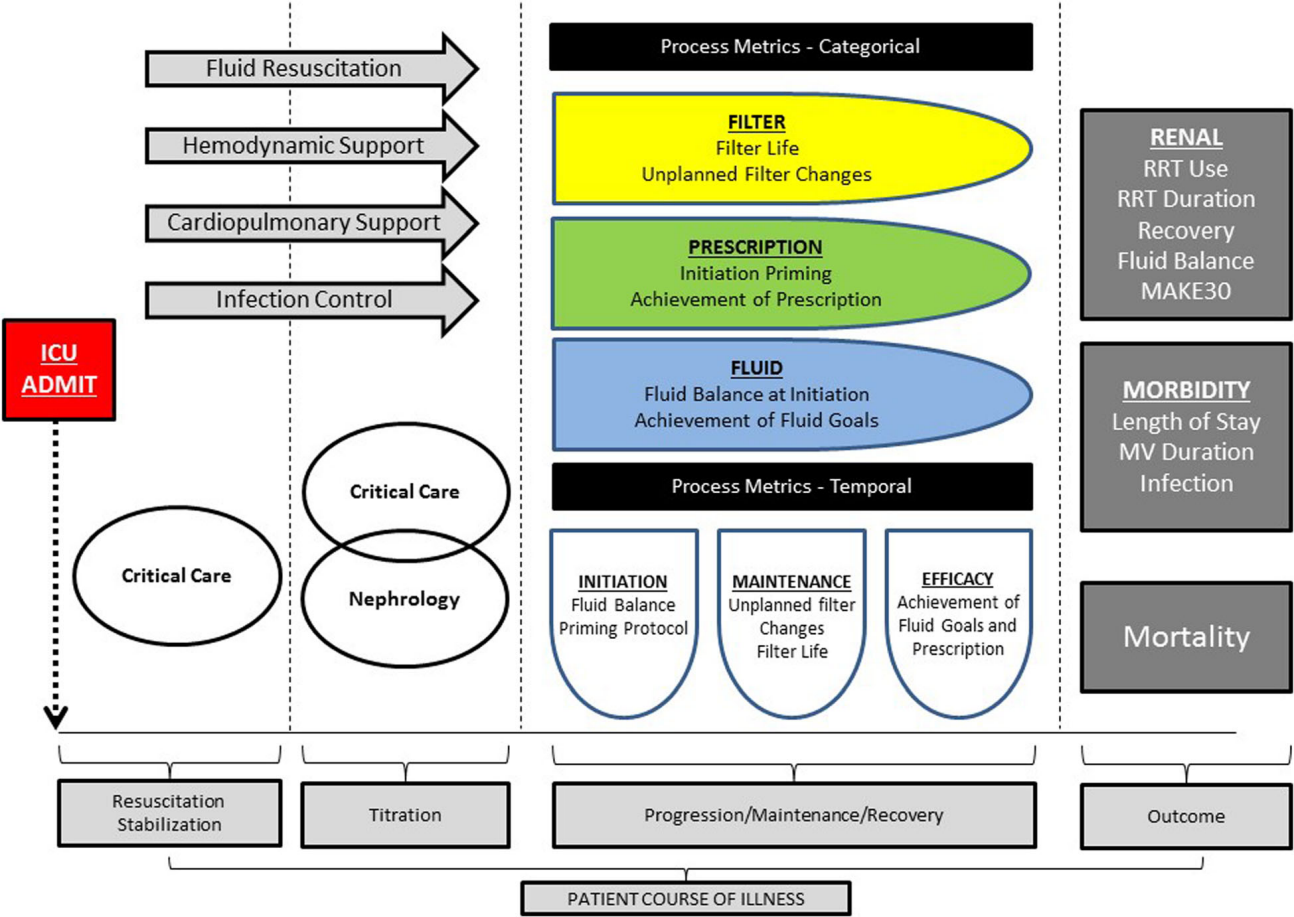
Process metrics in the Delivery of RRT. We believe that a proper analysis and objective quantification of the RRT delivery process will not only facilitate standards for centers and between institutions, but also provides the needed assurance and foundation that the afore mentioned “controversies” can be appropriately studied. RRT delivery quality can be quantified, studied, and compared. Ultimately, high quality RRT relies on these processes and needs to be assured if understanding how the technical factors associate with patient outcomes. Additionally, the essential outcome of “performance” can be added to the rubric of optimal RRT therapy. (RRT = renal replacement therapy, MAKE30 = major adverse kidney events in 30 days, MV = mechanical ventilation)

Many aspects of RRT delivery are controversial. Despite decades of study, the optimal dose of RRT is not known. Several recent studies investigating the optimal timing of initiation resulted in discordant findings (although the studies were significantly different in design [30–32]. In addition, many variables assumed to be associated with outcomes have been studied in some fashion or are currently actively being studied (Fig. 3). These metrics, however, are reliant on high quality, highly reproducible delivery of RRT. Therefore, how RRT is delivered matters and tracking RRT delivery process metrics can improve the quality of care delivery. We demonstrate an increase over time in filter life, a decrease in unplanned filter changes, and an increase in achievement of daily fluid goals. With the increased utilization of RRT during this time, a highly complex extracorporeal support modality, our survival remained constant. We conclude from this that our tracking process performance and adhering to benchmarks of process quality ensured that our “primary outcome” of survival remained constant, even amidst the high volume and the changing landscape of the traditional variables studied in RRT (Fig. 3). We believe that the process metrics of RRT delivery are the foundation of therapy and must be tracked if studied investigating how modifiable RRT practice parameters (timing of initiation, optimal dose, method of anticoagulation) affect patient outcome can actually be trusted (Fig. 4). A consistent, objective, and longitudinal assessment of performance is a standard of care.

Efforts to reduce practice variation in the intensive care unit (ICU) are underway. Individual practice variations can be an indicator of poor quality of care. Attention to the quality of cardiopulmonary resuscitation (CPR) for patients suffering cardiac arrest is an apt example. Data now indicates that survival from cardiac arrest is not simply related to the timely provision of CPR in response to an arrest, but the *quality* of the CPR itself [33]. For decades, time to CPR and time to defibrillation were believed to be the primary criterion associated with patient survival. The quality of CPR and the quality of defibrillation were assumed. Recent attention has now underscored the importance of pace and depth of compressions as markers of CPR quality. Subsequently, adherence to American Heart Association (AHA) guidelines dictating the depth and pace of compressions, and enhancing team cohesion via team training, has significantly improved survival [34]. Applying these same quality improvement strategies to CRRT care, by establishing measurable criteria to gauge CRRT care delivery, may enable an objective standard for evaluating performance, quality, and safety.

In total, the process measures we describe are central to the efficacious delivery of CRRT to a patient. Each measure objectively assesses an individual aspect of CRRT and together give us an illustrative and informative assessment of the state of our practice. Use of this CRRT process dashboard allows us readily to create internal standards of care, making it possible to quickly identify our own inconsistencies and spend time attempting to understand the reasons behind variability in practice. Ultimately, these process metrics are measurable and valuable to study in and of themselves but are likely directly impactful to the traditional hard patient outcomes specific to the kidney, to morbidity, and to mortality.

Our study has several limitations. As this is a single center study, the metrics and benchmarks are based on our institutional protocols and standard practice guidelines. While we demonstrate improvement in specific quality improvement measures, single center data is difficult to extrapolate to other centers as it is impacted by institutional practices. Additionally, the improvements in the delivery of CRRT care may not correlate with improving patient outcomes. Further studies are necessary to evaluate and better understand the impact of these quality improvement measures on the delivery of CRRT care.

## Conclusions

Continuous renal replacement is a highly complex and technical therapy delivered to critically ill patients. Assumptions about how effective the therapy is cannot be made simply by whether a patient survives. The process by which the therapy delivered is important and can be measured and, in reality, needs to be assured if the results of large scale trials investigating timing, anticoagulation, modality, and dose are to be trusted. Currently, very few studies describe efforts to improve the quality of CRRT care or even measure the performance of care delivery. We believe our standard for CRRT process measurement will facilitate the creation of standards, quality of care, and best practice guidelines by which benchmarking can be enabled – both within an institution and between institutions. The care for patients receiving CRRT can subsequently improve. Moving the needle of practice in an objective and measurable way towards consistency and mitigation of avoidable variability will improve safety for patients and the quality of CRRT care.

## Abbreviations

% FO: Percent fluid overload
AHA: American Heart Association
AKI: Acute kidney injury
CICU: Cardiac intensive care unit
CPR: Cardiopulmonary resuscitation
CRRT: Continuous renal replacement therapy
ECMO: Extracorporeal membrane oxygenation
FY: Fiscal year
ICU: Intensive care unit
NICU: Neonatal intensive care unit
PFC: Planned filter changes
PICU: Pediatric intensive care unit
ppCRRT: Prospective pediatric CRRT registry
UFC: Unplanned filter changes
